# Safeguarding Lysosomal Homeostasis by DNAJC5/CSPα-Mediated Unconventional Protein Secretion and Endosomal Microautophagy

**DOI:** 10.3389/fcell.2022.906453

**Published:** 2022-05-10

**Authors:** Juhyung Lee, Yue Xu, Yihong Ye

**Affiliations:** Laboratory of Molecular Biology, National Institute of Diabetes and Digestive and Kidney Diseases, National Institutes of Health, Bethesda, MD, United States

**Keywords:** DNAJC5/CSPα, cysteine string protein, ceroid lipofuscinosis neuronal, lysosome, endosomal microautophagy, misfolding-associated protein secretion (MAPS), protein quality control, unconventional protein secretion

## Abstract

Neuronal ceroid lipofuscinosis (NCL) is a collection of genetically inherited neurological disorders characterized by vision loss, seizure, brain death, and premature lethality. At the cellular level, a key pathologic hallmark of NCL is the build-up of autofluorescent storage materials (AFSM) in lysosomes of both neurons and non-neuronal cells. Molecular dissection of the genetic lesions underlying NCLs has shed significant insights into how disruption of lysosomal homeostasis may lead to lipofuscin accumulation and NCLs. Intriguingly, recent studies on DNAJC5/CSPα, a membrane associated HSC70 co-chaperone, have unexpectedly linked lipofuscin accumulation to two intimately coupled protein quality control processes at endolysosomes. This review discusses how deregulation of unconventional protein secretion and endosomal microautophagy (eMI) contributes to lipofuscin accumulation and neurodegeneration.

## Introduction

Neuronal Ceroid Lipofuscinosis (NCL, also named Batten diseases) refers to a group of genetically inherited lysosomal storage diseases that impact primarily neuronal functions in the central nervous system (Mole and Cotman, 2015). The diseases are rare with incidence rates varying from 1:14,000 to 1:1,000,000 depending on the geographic region (Williams, 2011). The diseases mostly affect infants and juveniles, although adult onset NCLs (ANCL) were recently reported. As expected, the infantile and juvenile forms (INCL and JNCL) are more severe, often associated with vision loss, seizure, dementia, and premature death at young ages (Cotman et al., 2013). By contrast, ANCL has relatively milder symptoms. Nevertheless, ANCL patients usually die within 10 years after diagnosis (Naseri et al., 2021).

At the cellular level, NCL is associated with progressive accumulation of autofluorescent lipopigments (lipofuscin) in both neurons and non-neuronal tissues (Haltia, 2006; Anderson et al., 2013; Naseri et al., 2021). These lipopigments appear to originate from endolysosomes as they often bear proteins of either endosomes or lysosomes. Lipid analysis has identified free fatty acids such as palmitic acid and arachidonic acid as the major lipid component in lipofuscin, which may result from increased phospholipase activities and/or abnormal membrane trafficking (Bazan et al., 1990).

To date, 13 types of NCLs have been clinically characterized (Table 1). While most NCL cases (those known as Batten diseases) are autosomal recessive, an autosomal dominant form of NCL referred to as Kufs disease was recently reported (Naseri et al., 2021). Genetic studies have identified many NCL-associated genetic mutations (Cotman et al., 2013; Specchio et al., 2020; Mole and Cotman, 2015) (Table 1). While most of the identified genes are linked to either INCL or JNCL, several ANCL-associated mutations have been found in *DNAJC5*, *CLN5*, *GRN*, and *CTSF* genes (Table 1). These genes, designated as CLNs (for ceroid lipofuscinosis neuronal), mostly encode proteins that regulate either lysosome dependent protein processing (e.g. PPT1 and CTSD) (Cotman et al., 2013) or the trafficking of lysosomal resident proteins (e.g. CLN6 and CLN8) (Bajaj et al., 2020; di Ronza et al., 2018). These findings further strengthen the tie between lipofuscin accumulation and endolysosomes, suggesting that neurodegeneration in NCLs may result from a deregulation in endolysosome homeostasis.

**TABLE 1:** A list of genes associated with various forms of CLN. Please add a reference column.

Human Disease	Gene	Protein	Protein Localization	Protein Function
CLN1	*PPT1* Vesa et al. (1995)	Palmitoyl-protein thioesterase 1	Lysosome	Protein localization regulation Gorenberg et al. (2021)
CLN2	*TPP1* Sleat et al. (1997)	Tripeptidyl-peptidase 1	Lysosome	Lysosomal protease Lin et al. (2001)
CLN3	*CLN3* Mitchison et al. (1997)	Battenin	Endolysosome	Lysosomal acidification Pearce et al. (1999)
CLN4	*DNAJC5* Nosková et al. (2011)	CSPα/DNAJC5	Endolysosome	Co-chaperone Braun et al. (1996)
CLN5	*CLN5* Savukoski et al. (1998)	CLN5	Lysosome	Lysosome to TGN trafficking Mamo et al. (2012)
CLN6	*CLN6* Gao et al. (2002)	CLN6	ER	Cargo trafficking Bajaj et al. (2020)
CLN7	*MFSD8* Siintola et al. (2007)	MFSD8	Lysosome	Transporter Sharifi et al. (2010)
CLN8	*CLN8* Ranta et al. (1999)	CLN8	ER	Cargo trafficking di Ronza et al. (2018)
CLN10	*CTSD* Siintola et al. (2006)	Cathepsin D	Lysosome	Lysosomal protease Cullen et al. (2009)
CLN11	*GRN* Smith et al. (2012)	Granulin	Lysosome	Lysosomal regulation Kao et al. (2017)
CLN12	*ATP13A2* Bras et al., (2012)	ATP13A2	Endolysosome	Polyamine transporter van Veen et al. (2020)
CLN13	*CTSF* Smith et al., (2013)	Cathepsin F	Lysosome	Lysosomal protease Shi et al. (2000)
CLN14	*KCTD7* Staropoli et al., (2012)	KCTD7	Cytosol	Unknown

### Lysosome Homeostasis Regulation

Lysosomes have long been recognized as critical metabolic compartments that break down not only proteins but also lipids, which make them a central hub of cellular homeostasis regulation (Pillay et al., 2002). Lysosomes receive proteins and lipids via both vesicular and non-vesicular trafficking routes. For example, lysosomes can fuse with vesicles originated from either the trans-Golgi network or the plasma membrane. While Golgi-derived vesicles deliver most lysosomal resident proteins, plasma membrane-originated vesicles are responsible for targeting cell surface molecules for lysosomal degradation. Under stress conditions (e.g., amino acid starvation), autophagy, a collection of “self-eating” mechanisms including macroautophagy, microautophagy, and chaperone-mediated autophagy (CMA) are activated, which recycle unwanted proteins to re-sculp the cellular proteome. Macroautophagy uses autophagosomes, a double membrane-encircled structure, to degrade cytosolic proteins as well as damaged or unwanted organelles such as endoplasmic reticulum (ER) and mitochondria (Dikic and Elazar, 2018). By contrast, microautophagy and CMA do not involve any vesicle intermediates. Instead, microautophagy moves cytosolic proteins or endosomal membranes into the lumen of late endosomes via inward membrane invagination, while CMA is believed to translocate cargos directly across the lysosomal membrane with the assistance of an oligomerized type I membrane protein named LAMP2A (Tekirdag and Cuervo, 2018; Fleming et al., 2022)

### Lysosome Biogenesis and Lysosomal Secretion

Given the essential role of lysosomes in protein homeostasis regulation, eukaryotic cells have adopted a conserved strategy to fine-tune the lysosomal degradation capacity in response to “lysosomal stress” conditions. A central regulator in this process is the transcription factor EB (TFEB), which under normal conditions, is phosphorylated by lysosome-associated kinase mTORC1 (Martina et al., 2012). Phosphorylated TFEB is sequestered in the cytosol in an inactive form due to association with scaffolding proteins of the YWHA (14-3-3) family. Under stress conditions such as amino acid starvation, ER stress etc., mTORC1 is released from lysosomes, causing dephosphorylation of TFEB. Dephosphorylated TFEB is then dissociated from YWHA and translocated into the nucleus to activate genes involved in lysosome biogenesis (Settembre et al., 2011).

Besides lysosome biogenesis, stressed cells can also activate another process termed lysosomal secretion or lysosomal exocytosis. In this process, lysosomes fuse with the plasma membrane to release luminal contents. This mechanism is thought to “purge” lysosomes of undegradable contents, and therefore “rejuvenate” stressed lysosomes. In a multicellular organism like humans, proteins released by lysosomal exocytosis may be internalized and degraded by cells specialized in “garbage-processing” such as macrophages.

Lysosomal secretion was first reported by Gilbert Vaes in 1968. While studying bone resorption, he observed that several acid hydrolases of lysosomes were released into the medium to catalyze bone absorption (Vaes, 1968). This phenomenon was later confirmed by other studies (Lee and Ye, 2018). In 1972, Miklos Muller showed that the release of hydrolases from *T. pyriformis* was caused by active secretion from what appears to be a special population of “lysosomes”, thus for the first time linking lysosomes to a secretory process (Müller, 1972). Subsequent studies showed that upon activation by calcium, cytotoxic T cells and natural killer cells could release cytolytic proteins that had been stored in secretory granules, which shared features of lysosomes as they contained hydrolytic enzymes and lysosomal membrane proteins (Blott and Griffiths, 2002). Subsequent work by Andrews and colleagues showed that lysosomal secretion was tightly regulated in many cell types including fibroblast, myoblast and epithelial cells (Rodríguez et al., 1997; Jaiswal et al., 2002). The precise mechanism underlying lysosomal secretion is unclear. Several studies have implicated a GTP-dependent step involving the ADP-ribosylation factor 1 (ARF1), phospholipase D, and a phosphatidylinositol transfer protein (PITP) in lysosomal secretion (Stutchfield and Cockcroft, 1993; Fensome et al., 1996; Jones et al., 1999), but how these factors act in concert to facilitate lysosomal exocytosis is unknown. Importantly, it remains to be demonstrated whether lysosomal secretion occurs at mature degradation-competent lysosomes or at a pre-lysosomal compartment originated from the Golgi system, which still retains secretory capacity (Borland and Vilhardt, 2017).

### Lysosome Repair and Lysophagy

When the integrity of the endolysosomal membrane is damaged, a membrane repairing pathway is activated, which was revealed recently with the application of a lysosomotropic dipeptide, L-leucyl-L-leucine methyl ester (LLOMe) (Thiele and Lipsky, 1990). When cells are treated with LLOMe, it is rapidly internalized into endolysosomes. In this acidic environment, LLOMe is condensed into small crystals that can permeabilize the endolysosomal membrane. This results in the rapid recruitment of endosomal sorting complex required for transport (ESCRT) proteins to endolysosomes (Radulovic et al., 2018; Skowyra et al., 2018). ESCRT complexes (0, I, II, and III) were initially identified as key regulators that control the sorting of endosomal membrane and cytosolic cargos via the so called multivesicular body pathway in *S. cerevisiae*. These complexes act in sequential order to recruit ubiquitinated cargos to the vacuole or lysosome surface, driving the inward budding of membranes to form intralumenal vesicles (Katzmann et al., 2001; Shields et al., 2009). Additional studies have attributed several topologically related functions to ESCRTs including cytokinesis, viral budding, plasma membrane repair (Vietri et al., 2020). In the lysosome repairing pathway, the recruitment of ESCRTs to endolysosomes is triggered by calcium efflux from damaged lysosomes, which activates ALIX, a lipid binding component of the ESCRTs (Skowyra et al., 2018). Recruited ESCRTs may serve as patches to temporarily seal damaged membranes, but permanent removal of the damaged membrane may require the budding of membranes into the lumen of endolysosomes, which is driven by the assembly of the filamentous ESCRT III complex.

When damages to lysosomes are too severe to be repaired, cells use a specialized macroautophagy mechanism termed lysophagy to remove damaged lysosomes (Papadopoulos et al., 2020). Unlike lysosome repair, lysophagy was triggered by the exposure of glycans in certain glycoproteins that normally reside only in the lumen of lysosomes (Jia et al., 2020). Given the size of these proteins and the bulky glycans attached, it is generally assumed that the exposure of these glycans on the surface of lysosomes would require either a full rupture or damages that are big enough to allow the movement of these proteins across the lysosomal membrane. The exposed glycans can be sensed by a group of cytosolic lectins named Galectin, which in turn recruits ubiquitination machinery such as the E3 ubiquitin ligase TRIM16 (Chauhan et al., 2016). Alternatively, exposed glycans may directly recruit certain ubiquitin ligases that have a glycan-binding activity (e.g., FBXO27) (Yoshida et al., 2017). Additionally, a recent study identified UBE2QL1, a ubiquitin conjugating enzyme (E2) as a critical regulator of lysophagy (Koerver et al., 2019). The recruitment of these ubiquitination factors led to massive ubiquitination of proteins on damaged endolysosomes, which then further engage downstream effectors such as the AAA (ATPase associated with diverse cellular activities) ATPase VCP to clear damaged lysosomes.

### DNAJC5/CSPα is Membrane-Associated Protein That has a Neuroprotective Function

How can deregulation in lysosome homeostasis cause NCL? The answer to this question is poorly understood, but recent genetic and biochemical studies on a HSC70/HSP70 co-chaperone named DNAJC5/CSPα have provided some important clues.

CSPα (also named as DNAJC5 or CLN4) is a member of the HSP40 co-chaperone family that serves as a cofactor for the major heat shock protein HSC70/HSP70. Like other HSP40 family members, CSPα can stimulate the ATPase activity of HSC70/HSP70 (Braun et al., 1996; Russell et al., 1999). In addition to *DNAJC5/CSPα*, the human genome also contains two other CSPα-related genes, *CSPβ* and *CSPγ*. The encoded proteins share ∼80% similarity with CSPα. Because the expression and function of CSPβ and CSPγ appear to be restricted to the testis (Fernández-Chacón et al., 2004; Gorleku and Chamberlain, 2010), we focus our discussions on CSPα in this review.

**FIGURE 1:**
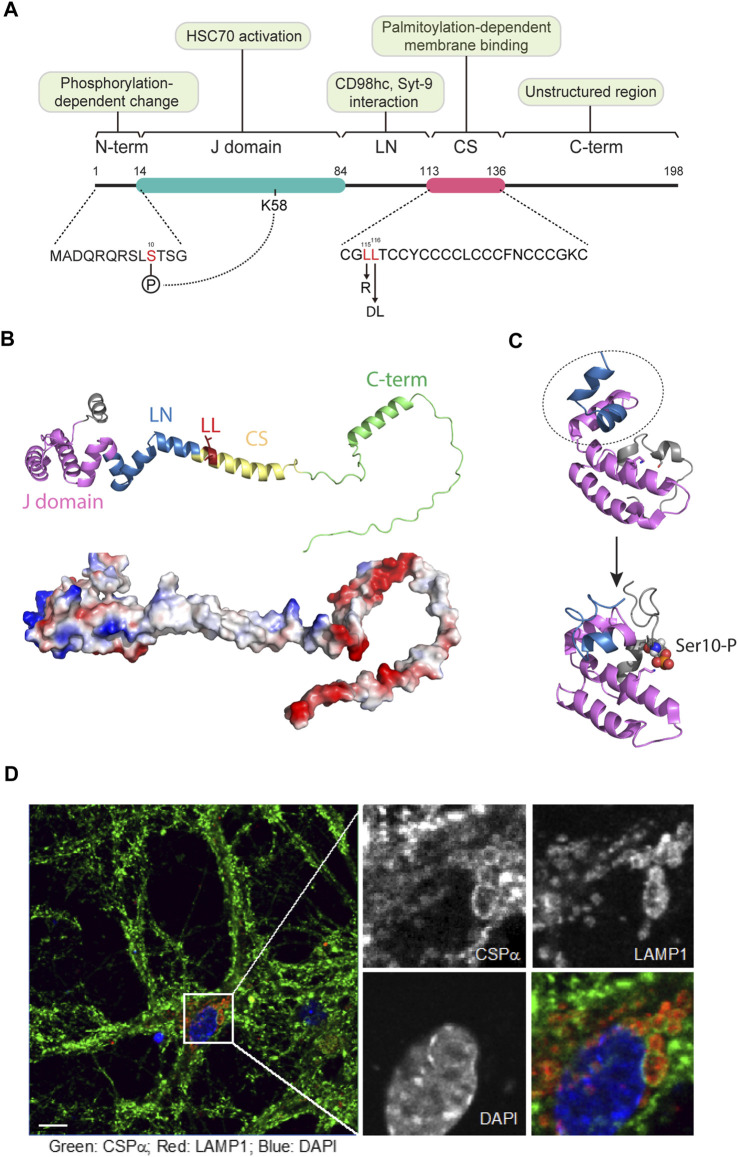
The structure and subcellular localization of CSPα. **(A)** The domain structure of CSPα. CSPα consists of 5 domains, a small N-terminal (N-term.) segment, a DnaJ (J) domain, a hydrophobic linker (LN), a cysteine string (CS) domain, and a disordered C-term domain. Phosphorylation at Ser10 in the N-domain by PKA or PKB may allow CSPα activation by forming an intramolecular interaction between p-Ser10 and Lys58 in the J domain. The conserved J domain is essential for HSC70 interaction and activation. The LN domain can interact with other proteins such as Synaptotagmin-9 and CD98hc, which regulate SNARE complex assembly and MAPS, respectively. The CS domain possesses 14 cysteine residues for palmitoylation, engaging CSPα to membrane compartments. Mutations in two leucine residues (L115R and ΔL116) within the CS domain are linked to ANCL disease. **(B)** Upper panel, A ribbon model of full length human CSPα predicted by Alphafold (Identifier, AF-Q9H3Z4-F1). Each domain is labeled in colors. N-terminal domain, grey; J domain, pink; LN domain, blue; cysteine-string, yellow; C-terminal domain, light green. The ANCL-linked mutations in the CS domain are highlighted in red. Lower panel, a surface electrostatic potential view of the CSPα Alphafold model. **(C)** A phosphorylation dependent conformational change in the CSPα J domain as revealed by NMR. PDB: 2N04 and 2N05. Notice that the subdomain labeled in dashed oval rotates down to pack on the other subdomain labeled in magenta when Ser10 is phosphorylated. **(D)** The subcellular localization of CSPα in primary neurons. Murine primary hippocampal neurons at DIV10 were stained by antibodies for CSPα (green) and the lysosomal marker LAMP1 (red). Note that CSPα in Soma is localized to vesicular structures that overlap with LAMP1. Nuclei were labeled by DAPI in blue. Scale bars, 10 µm.

Human CSPα encodes a 198 amino-acid long polypeptide that contains three conserved domains: an amino-terminal (N) HSC70-binding J-domain, a central cysteine string (CS) domain, and a linker (LN) domain between the J- and the CS domains (Chamberlain and Burgoyne, 2000) (Figure 1A). Additionally, CSPα also contains a relatively long C-terminal segment that is predicted to be largely unstructured, and a small N-terminal segment preceding the J-domain (Figure 1B). The latter contains several putative phosphorylation sites that may regulate CSPα activities (Figure 1C) (see below). The cysteine residues in the CS domain are known to undergo palmitoylation (Greaves and Chamberlain, 2006; Greaves et al., 2008). Several palmitoyl transferases are capable of palmitoylating CSPα when overexpressed, but DHHC5/HIP14 appears to be the major one responsible for proper membrane localization of endogenous CSPα (Ohyama et al., 2007; Stowers and Isacoff, 2007). CSPα palmitoylation can be reversed by the action of PPT1 (Henderson et al., 2016), a depalmitoylating enzyme encoded by the *CLN1* gene. The N terminal J-domain consists of four α-helices, which are packed into a tightly folded domain. It contains a highly conserved histidine-, proline-, and aspartic acid-containing motif (HPD), which is crucial for the HSC70/HSP70 binding and ATPase-stimulating activities (Jiang et al., 2007).

Two mutations in *DNAJC5* are associated with ANCL (Noskova et al., 2011; Benitez et al., 2011; Cadieux-Dion et al., 2013). These mutations result in either a substitution of Leu115 to Arg (L115R) or the deletion of Leu116 (ΔL116), both of which are located within the CS domain (Figures 1A,B). Recent studies suggest that these mutations reduce CSPα palmitoylation while increasing its aggregation propensity (Benitez and Sands, 2017; Diez-Ardanuy et al., 2017; Imler et al., 2019; Naseri et al., 2020). Additionally, these mutations cause the mis-localization of the mutant proteins in cells (Imler et al., 2019). Accordingly, ANCL-associated *DNAJC5* mutations are thought to reduce the CSPα chaperoning function (Naseri et al., 2020).

*DNAJC5* is widely expressed in a variety of human tissues (Coppola and Gundersen, 1996). In neurons, CSPα is mainly detected on synaptic vesicles at the presynaptic terminal (Zinsmaier et al., 1990; Ohyama et al., 2007; Tobaben et al., 2001), but a fraction was also seen on lysosomes (Figure 1D) (Benitez and Sands, 2017). In non-neuronal cells, CSPα is more prominently localized to late endosomes/lysosomes with a fraction detected in a peri-nuclear compartment and some on the cell surface (Xu et al., 2018; Lee et al., 2022).

Genetic studies in mice and model organisms such as fruit flies have suggested a neuroprotective role for CSPα. *D. Melanogaster* has only one *CSP* gene and its inactivation results in embryonic lethality with a small percent of flies (<5%) surviving to adult stage. These escapers bear a variety of neurological phenotypes including sluggishness, uncoordinated movement, and premature death (Zinsmaier et al., 1990; Burgoyne and Morgan, 2015). Surprisingly, CSPα knockout mice are viable at birth, but these mice usually suffer age-dependent synapse loss and massive neurodegeneration, particularly in the retina. These mice usually die at 8 weeks of age (Fernandez-Chacon et al., 2004; Schmitz et al., 2006; Garcia-Junco-Clemente et al., 2010). Primary neurons isolated from CSPα knockout mice also undergo neurodegeneration *in vitro* (Garcia-Junco-Clemente et al., 2010). These observations have unambiguously established an essential role for CSPα in neuronal development.

## Molecular Functions of DNAJC5/CSPα

### A Chaperoning Function in Membrane Trafficking

How does inactivation of *DNAJC5/CSP* cause the above-mentioned phenotypes? Early studies in flies suggested that neurodegeneration might be caused by a defect in calcium-elicited neurotransmitter release (Umbach et al., 1994; Zinsmaier, 2010). This finding, together with the reported interaction of CSPα with membrane fusion regulators such as synaptobrevin and synaptotagmin, prompted the idea that CSPα may regulate exocytosis by modulating the stability/activity of these SNARE proteins (Evans and Morgan, 2002; Boal et al., 2004).

Given the well-established role of CSPα as a HSC70/HSP70 co-chaperone, significant efforts were made in search of CSPα substrates. Presumably, substrates should associate with CSPα either directly or indirectly and they should either accumulate in an unfolded state or undergo rapid degradation by a protein quality control mechanism in CSPα deficient cells. Protein binding analyses suggested several candidate substrates including VAMP-1, G-protein subunits, SNAP25, and N-type calcium channels (Chamberlain et al., 2001). Among them, SNAP25 is a synaptic SNARE protein that has been extensively characterized. SNAP25 interacts with CSPα via HSC70 and is subject to ubiquitination and proteasomal degradation in CSPα deficient cells (Chandra et al., 2005; Sharma et al., 2011). Lentivirus-mediated overexpression of SNAP25 rescued neurodegeneration in CSPα deficient animals, confirming it as a mediator of cell death in CSPα null neurons (Sharma et al., 2012). Since SNAP25 is a component of a t-SNARE complex that mediates membrane fusion in exocytosis, its downregulation in CSPα knockout neurons offers a seemingly straightforward explanation for the neurotransmission defect in CSPα deficient animals. However, an alternative explanation was proposed when subsequent studies identified a vesicle recycling defect in CSPα deficient cells, which was attributed to deregulation of another CSPα substrate, the endocytic GTPase Dynamin-1 (Rozas et al., 2012; Zhang et al., 2012). CSPα not only maintains the stability of Dynamin-1 but also promotes its oligomerization during endocytosis. These findings raise the possibility that CSPα may couple exocytosis to endocytosis to ensure efficient synaptic vesicle recycling (Gross and von Gersdorff, 2016). Thus, defects in exocytosis may be secondary due to lack of endocytosis, which leads to a depletion of synaptic vesicles.

Intriguingly, neurodegeneration associated with CSPα depletion can be at least in part rescued by overexpression of α-synuclein (α-syn) (Chandra et al., 2005), another synaptic vesicle-associated protein well known for its presence in Lewy bodies in Parkinson disease (Spillantini et al., 1997). Moreover, genetic mutations or gene duplication in the α-syn-encoding gene *SCNA* are linked to a familial form of Parkinson disease (Stefanis, 2012). Although α-syn has been subject to extensive study, its physiological function remains poorly understood. The genetic interaction of *SCNA* with *DNAJC5* suggests α-syn as a potential regulator of synaptic exocytosis or vesicle recycling. Since overexpression of α-syn does not rescue the SNAP25 downregulation phenotype in CSPα knockout animals, it may act downstream or in parallel to SNAP25 in membrane trafficking.

### Eliminating Misfolded Proteins via MAPS

Protein misfolding imposes a major threat to cell homeostasis because misfolded proteins are not only defective in functions but also prone to aggregation. To cope with protein misfolding-associated proteotoxic stress, eukaryotic cells have evolved a variety of protein quality control (PQC) mechanisms, which include the ubiquitin-proteasome system, macroautophagy, microautophagy and CMA. Many chaperones such as HSC70/HSP70 and members of the HSP40 family play pivotal roles in these processes. Intriguingly, recent studies have underscored an unexpected PQC mechanism that exports misfolded proteins to the cell exterior by CSPα-assisted unconventional protein secretion (Fontaine et al., 2016; Xu et al., 2018; Lee et al., 2022; Wu et al., 2022).

Unconventional protein secretion refers to a collection of protein trafficking mechanisms that either export proteins lacking an endoplasmic reticulum (ER)-targeting signal sequence or transport proteins from the ER to the cell surface independent of the Golgi system (Nickel and Rabouille, 2009; Malhotra, 2013; Zhang and Schekman, 2013). To date, only a handful of unconventional secretion substrates have been characterized, which include FGF2, IL1β, α-syn, and Tau etc. but the list of unconventional secretion substrates is rapidly expanding. Unconventional secretion cargos can use a vesicle intermediate to reach the cell exterior (Rabouille, 2017), or in the case of FGF2 and Tau, direct translocation across the plasma membrane has been reported (Steringer et al., 2017; Katsinelos et al., 2018; Merezhko et al., 2018). Although many unconventional secretion cargos characterized to date are released in a native form to exert their functions in the extracellular environment, our recent work suggested that higher eukaryotic cells can also release misfolded cytosolic proteins via a secretion mechanism termed as misfolding-associated protein secretion (MAPS) (Lee et al., 2016).

MAPS was discovered serendipitously while we characterized an ER-associated deubiquitinase named USP19, which also harbors a chaperone activity and a C-terminal transmembrane domain (Lee et al., 2016). Biochemical study showed that USP19 binds to two major heat shock proteins HSC70 and HSP90 in cells, suggesting a possible role in PQC (Lee et al., 2014). Although the localization of USP19 to the ER suggested a possible function in ER-associated protein degradation (ERAD), this model has not been conclusively established. Instead, we found that USP19 overexpression promoted the release of certain cytosolic proteins while its inactivation inhibited unconventional protein secretion in mammalian cells. In this regard, USP19 preferentially promotes the secretion of misfolded proteins such as engineered mutant proteins, unassembled protein subunits, and some wild-type proteins that are prone to misfolding such as Tau and α-syn, which are known contributors to Alzheimer and Parkinson diseases, respectively. Many MAPS substrates are also subject to degradation by the ubiquitin-proteasome system. Thus, it appears that MAPS may act as a supplementary protein quality control mechanism to enhance the clearance of misfolded proteins. Consistent with this model, USP19 deficient cells are more sensitive to proteasome inhibitor-induced cytotoxicity (Lee et al., 2016).

Many neurodegenerative disease-associated MAPS substrates are also known to bind to HSC70 and/or CSPα. Consistent with this finding, Fontaine et al. showed that CSPα could act together with HSC70 to promote the release of Tau, TDP-13, and α-syn from both non-neuronal cells and neurons (Fontaine et al., 2016). These disease-associated misfolded proteins were released largely in a free form, not associated with any extracellular vesicles (Lee et al., 2016). A subsequent study showed that both CSPα and HSC70 functioned downstream of USP19 to promote MAPS as knockdown of CSPα or HSC70 inhibited USP19-induced protein secretion (Xu et al., 2018).

**FIGURE 2:**
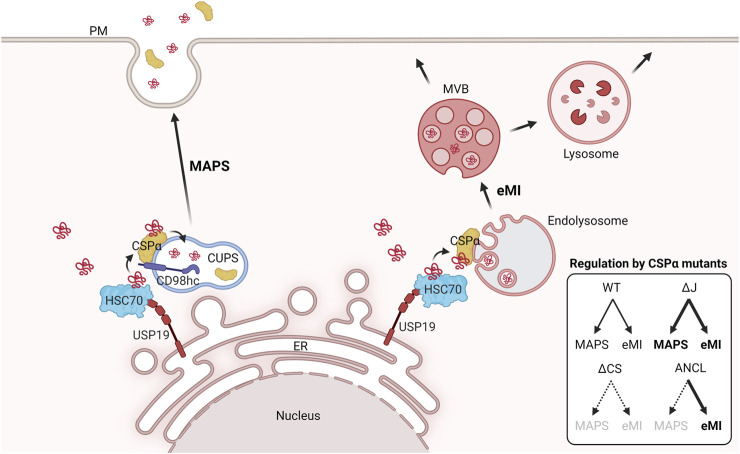
CSPα couples MAPS to eMI to promote lysosome homeostasis. In mammalian cells, CSPα triages cytosolic misfolded proteins by two different mechanisms: misfolding-associated protein secretion and eMI-mediated degradation. In both cases, an ER-associated deubiquitinase USP19 recruits and deubiquitinates misfolded substrates at the ER. For MAPS, CSPα and HSC70 guide the cargos to a peri-nuclear secretory compartment for unconventional protein secretion (CUPS). CD98hc is required for targeting of CSPα/substrate complexes to this compartment. After CSPα chaperones substrates to the lumen of CUPS, the encapsulated MAPS cargos and CSPα are secreted possibly by vesicular trafficking between the CUPS and plasma membrane (PM). For eMI, CSPα also escorts misfolded proteins into endolysosomes by an ESCRT-dependent mechanism. The resulting multivesicular bodies containing misfolded cargos can be degraded or secreted after the fusion of MVBs with lysosomes or plasma membrane. The box indicates how these two processes can be differentially regulated by different CSPα mutant proteins. The figure was created by BioRender.

How do cells secrete misfolded cytosolic proteins lacking a signal sequence? Several lines of evidence suggest that MAPS substrates probably use one or more vesicle carriers as a secretory intermediate compartment, and it is possible that for a given substrate like Tau, multiple secretion routes are involved. Several types of vesicles, endolysosomes in particular, have been suggested to function in unconventional secretion given the previously documented lysosomal exocytosis (see above) (Lee and Ye, 2018). In *S. cerevisiae*, a Golgi-derived membrane compartment termed CUPS (Compartment for Unconventional Protein Secretion) was reported as a major mediator for nitrogen starvation-induced unconventional protein secretion (Malhotra, 2013). Our recent work suggested a peri-nuclear membrane compartment in proximity to the Golgi system as a CUPS equivalent compartment in mammalian cells (Figure 2A). A fraction of CSPα is localized to this compartment, which is regulated by SLC3A2/CD58hc, a common adaptor for several amino acid transporters (Lee et al., 2022). The peri-nuclear CSPα appears to retrieve misfolded cargos from ER-localized USP19 and accompany them to the CUPS for secretion (Xu et al., 2018). As expected, the CS domain essential for palmitoylation is crucial for localizing CSPα to the peri-nuclear compartment and for MAPS (Xu et al., 2018; Lee et al., 2022). Importantly, CS-mediated palmitoylation appears to drive CSPα into a large oligomeric assembly, which stimulates protein secretion (Wu et al., 2022). It is noteworthy that neither USP19 nor CSPα is absolutely essential for MAPS because knockout of either of these genes only led to a partial defect in MAPS (Xu et al., 2018), suggesting functional redundancy with other membrane-associated chaperones or the existence of parallel secretion mechanisms.

How to translocate cargoes into the lumen of the CUPS is currently a major open question. A recent study using the unconventional secretion cargo IL1β as a bait identified a membrane protein named TMED10, which appeared to mediate protein translocation across the membrane in unconventional protein secretion (Zhang et al., 2020). TMED10 is a single-spanning membrane protein localized to the ERGIC (ER and Golgi intermediate compartment). It belongs to the EMP24/GP25L/p24 cargo receptor family, which is generally involved in ER to Golgi trafficking (Strating and Martens, 2009). Knockout of TMED10 reduced not only IL1β secretion but also the release of many other unconventional secretion cargos. *In vitro* reconstitution experiments suggested that TMED10 might facilitate cargo translocation into the lumen of a secretory compartment by binding to a consensus motif in cargos (Zhang et al., 2020). However, whether TMED10 forms a protein-conducting channel or uses other means to promote unconventional protein secretion remains to be elucidated.

### Protein Quality Control by Endosomal Microautophagy

Endosomal microautophagy (eMI) refers to a special form of autophagy in which late endosomes or lysosomes take up cytoplasmic materials by membrane invagination and pinching off, forming multivesicular bodies (MVBs) (also called intralumenal vesicles) (Marzella et al., 1981; Oku and Sakai, 2018). This process is conserved from *S. cerevisiae* to humans, involving several ESCRT machinery proteins (Zhang et al., 2021). Because MVB formation is coupled to the engulfment of a portion of the cytosol into late endosomes, which is then degraded together with the invaginated membranes by lysosomes, eMI-mediated protein and membrane turnover appears to be largely non-selective. However, recent studies have revealed several types of selective eMI in yeast, fruit flies and mammalian cells (Sahu et al., 2011; Mukherjee et al., 2016; Mejlvang et al., 2018; Lee et al., 2020; Lee et al., 2022).

Selective eMI was initially suggested when eMI cargos were found to undergo ubiquitination in yeast (Katzmann et al., 2001). Subsequent studies identified several ubiquitin binding motifs in ESCRT complexes (Shields et al., 2009), which function in cargo selection and recruitment (MacDonald et al., 2012). Selective eMI was later confirmed in mammalian cells (Sahu et al., 2011). Using an *in vitro* reconstitution system, Sahu and colleagues demonstrated that cytosolic proteins bearing a KFERQ-containing motif could be directly translocated into late endosomes in a LAMP2A independent but HSC70-, KFERQ-, and ESCRT-dependent manner. Further analyses suggested that HSC70 binds to eMI substrates and then uses a cationic domain to associate with endosomal membrane phosphatidylserines, linking substrates to late endosomes (Morozova et al., 2016; Uytterhoeven et al., 2015). Interestingly, the KFERQ-motif has also been known to direct proteins to the CMA pathway. A recent study on Tau suggests that this misfolding-prone protein is constitutively degraded by CMA because of multiple KFERQ-like motifs. However, upon acetylation, Tau is rerouted to eMI for degradation or release by exosomal secretion (Caballero et al., 2021). In addition to KFERQ-dependent eMI, our recent study showed that CSPα also participated in selective eMI (Figure 2B) (Lee et al., 2022). In both neuron and non-neuronal cells, a fraction of CSPα is tightly associated with endolysosomal membranes. Intriguingly, despite the lack of the KFERQ motif, endolysosome-associated CSPα can efficiently enter into multivesicular bodies together with bound cargos (Lee et al., 2018). As expected, this process involves the ESCRT machinery, but surprisingly, is independent of the J domain of CSPα (Lee et al., 2022). How CSPα recruits substrates to endolysosomes and how it cooperates with HSC70 in eMI remain to be determined. Additionally, the role of CSPα palmitoylation in eMI also needs to be better defined.

### Regulation of MAPS and eMI

In general, the ubiquitin-proteasome system and macroautophagy degrade substrates quite efficiently. By contrast, MAPS appears to operate only at low capacity under normal conditions because both USP19 and CSPα contain an autoinhibitory domain that restricts their activities in this process. The autoinhibitory domain of USP19 is a UBL (ubiquitin like)-containing domain inserted in the middle of the USP (ubiquitin specific protease) domain (Xu et al., 2018). For CSPα, the autoinhibitory domain is the HSC70-binding J-domain (Lee et al., 2022). When these domains are removed, the resulting truncated proteins are significantly more activated than the wild-type counterpart in MAPS. These autoinhibitory mechanisms appear to be applicable to eMI as the J-domain deleted CSPα mutant is more efficiently translocated into endolysosomes than wild-type CSPα (Lee et al., 2022). These observations raise the possibility that these proteins may be activated under stress conditions to promote substrate flow to eMI. Consistent with this notion, eMI is indeed upregulated under the conditions of nutrient starvation, DNA damage, and oxidative stress, although whether this is achieved via activating USP19 or CSPα remains to be established (Mukherjee et al., 2016; Lee et al., 2020; Mesquita et al., 2020).

Thus, understanding the regulatory mechanism of USP19 and CSPα may provide some clues on when and how MAPS and CSPα-dependent eMI are activated. Due to limited structural information, the regulation of USP19 is poorly understood. However, our proteomic study identified HSC70 and HSP90 as two major binding partners of USP19 (Lee et al., 2014). We further showed that HSC70 but not HSP90 was required for USP19-mediated MAPS (Xu et al., 2018). These findings corroborate the idea that MAPS might be regulated by proteotoxic stress, a notion further supported by the finding that the secretion of misfolded proteins is generally upregulated in cells treated with proteasome inhibitors (Lee et al., 2016; Lee et al., 2005). For CSPα, NMR studies suggested that the J-domain, when phosphorylated at Ser10, was packed into a globular domain, but dephosphorylation disrupted the interdomain interaction (Figure 1C), resulting in a conformational change that may be essential for the function of CSPα (Patel et al., 2016).

Intriguingly, the MAPS and eMI pathways appear to be tightly coupled as conditions that increase eMI often stimulate MAPS as well. Therefore, for a long time, it was assumed that misfolded proteins might use endolysosomes as a secretory intermediate compartment in MAPS. However, several lines of evidence now suggests that these two processes are parallel mechanisms coupled by CSPα. First, while the J domain-deleted CSPα mutant has a much-increased activity in promoting α-syn secretion, it only modestly promotes the translocation of α-syn into endolysosomes. More importantly, a dominant negative VPS4 mutant that disrupts the function of the ESCRT III complex in eMI can increase the secretion of several MAPS substrates although it completely blocks the endosomal translocation of these proteins (Lee et al., 2022).

### DNAJC5/CSPα Dysfunction in ANCL

Although loss of CSPα function in animals causes neurodegeneration, the ANCL-associated CSPα mutations do not seem to act as a loss-of-function allele because lipofuscin accumulation, albeit being readily observed in cells overexpressing CSPα L115R or ΔL116 mutants (Naseri et al., 2020; Lee et al., 2022), has not been reported in CSPα deficient cells (Schmitz et al., 2006).

How do mutations in CSPα cause lipofuscin accumulation and neurodegeneration? Our recent study suggests that lipofuscin accumulation may be caused by abnormal membrane flow due to an imbalance between unconventional protein secretion and eMI (Lee et al., 2022). The fact that CSPα activation stimulates both MAPS and eMI suggests a necessity to couple these two quality control pathways, which conceivably may prevent the overflow of misfolded proteins and membranes into endolysosomes and thus inhibit lipofuscin biogenesis. Several lines of evidence indicate that inhibiting MAPS while maintaining eMI is sufficient to induce lipofuscin accumulation (Figure 2). First, both L115R and ΔL116 CSPα mutants are defective in MAPS (Lee et al., 2022; Wu et al., 2022). However, these mutants are capable of translocating into endolysosomes via eMI. Likewise, a CSP mutant lacking the linker domain is also defective in MAPS, but active in eMI, and overexpression of this mutant induces lipofuscin accumulation similarly as the disease-associated mutants (Lee et al., 2022). Finally, knockout of SLC3A2/CD98hc inhibits MAPS but does not significantly affect eMI. Intriguingly, a significant fraction of CD98hc deficient cells contain a single giant ‘lysobody’, which is a sphere-shaped autofluorescent organelle. This organelle is wrapped around by CSPα and late endosomal proteins such as LAMP1 and Rab9, indicating endolysosomes as its precursor. Given that many CLN-associated mutations known to date are recessive loss-of-function alleles that cause a deficiency in lysosomal degradation, our study underscores a special class of CLN mutations that cause abnormal flow of membranes and misfolded proteins into endolysosomes, which dominantly disrupts lysosomal function. Our model is also consistent with recent studies implicating several other CLN proteins in unconventional protein secretion (Huber, 2021).

## Conclusion

The implication of CSPα in eMI and unconventional protein secretion has significantly expanded the functional repertoire of CSPα, which establishes it as a key protein quality control regulator. These new findings, while providing new insights on the pathogenic mechanisms underlying NCL, also raise many questions pertaining to the role of endolysosomal trafficking in lipofuscin biogenesis. Most importantly, it would be important to gather more evidence to support the hypothesis that abnormal MAPS and eMI are a key contributor to neuronal lipofuscinosis and neuronal cell death. Given the specific lipid composition of the lipofuscin, it would be important to determine whether MVB formation in eMI has specific lipid requirement or involves specific lipases, which may lead to increased deposit of certain lipids in endolysosomes when this pathway is deregulated. Additionally, a thorough understanding of the physiological relevance of eMI requires a better characterization of the cellular mechanisms that activate eMI, particularly regarding how CSPα is regulated and what physiological eMI substrates are.
